# Differential effects of stimulus strength and volitional control in bistable perception

**DOI:** 10.1186/1471-2202-15-S1-P60

**Published:** 2014-07-21

**Authors:** James Rankin, John Rinzel

**Affiliations:** 1 Center for Neural Science, New York University, 4 Washington Place, 10003 New York, NY; 2 Courant Institute of Mathematical Sciences, New York University, 251 Mercer St, 10012 New York, NY

## 

Bistable perception has been widely studied in the visual system where ambiguity in sensory information coming from, for example, binocular, depth or motion cues leads to spontaneous shifts in perception. Indeed, modeling of rivalrous dynamics induced by ambiguity in these visual sensory cues led to a generalized Levelt’s proposition II that describes the effect of stimulus strength manipulations around equidominance: ``the mean dominance duration of the stronger percept changes more than that of the weaker percept'' [1]. In [2] it was shown that auditory and visual bistability share the common traits of perceptual bistability using ambiguous auditory streaming and visual motion stimuli. In each modality there are alternations between a grouped percept and a split percept. They further investigated the effect of volitional control at equidominance and found that attending to one percept (grouped or split) reduced mean dominance durations of the unattended (weaker) percept. These findings are incompatible with the generalized Levelt’s Proposition II if one assumes that volition increases the strength of the targeted percept. We propose a volitional mechanism with state-dependent inputs in order to resolve this apparent conflict.

We work with a widely used competition model (Fig 1A) for bistable dynamics that incorporates mutual inhibition and slow adaptation [3]. We incorporate input normalization as proposed in [1]. Symmetry is assumed between the competing percepts such that when input to each population is balanced the alternations generated by the model are at equidominance. When the input is increased to population #1, more time is spent with #1 active (Fig 1B). Furthermore, #1’s percept duration changes the most given more input, which is consistent with generalized Levelt's II.

**Figure 1:**
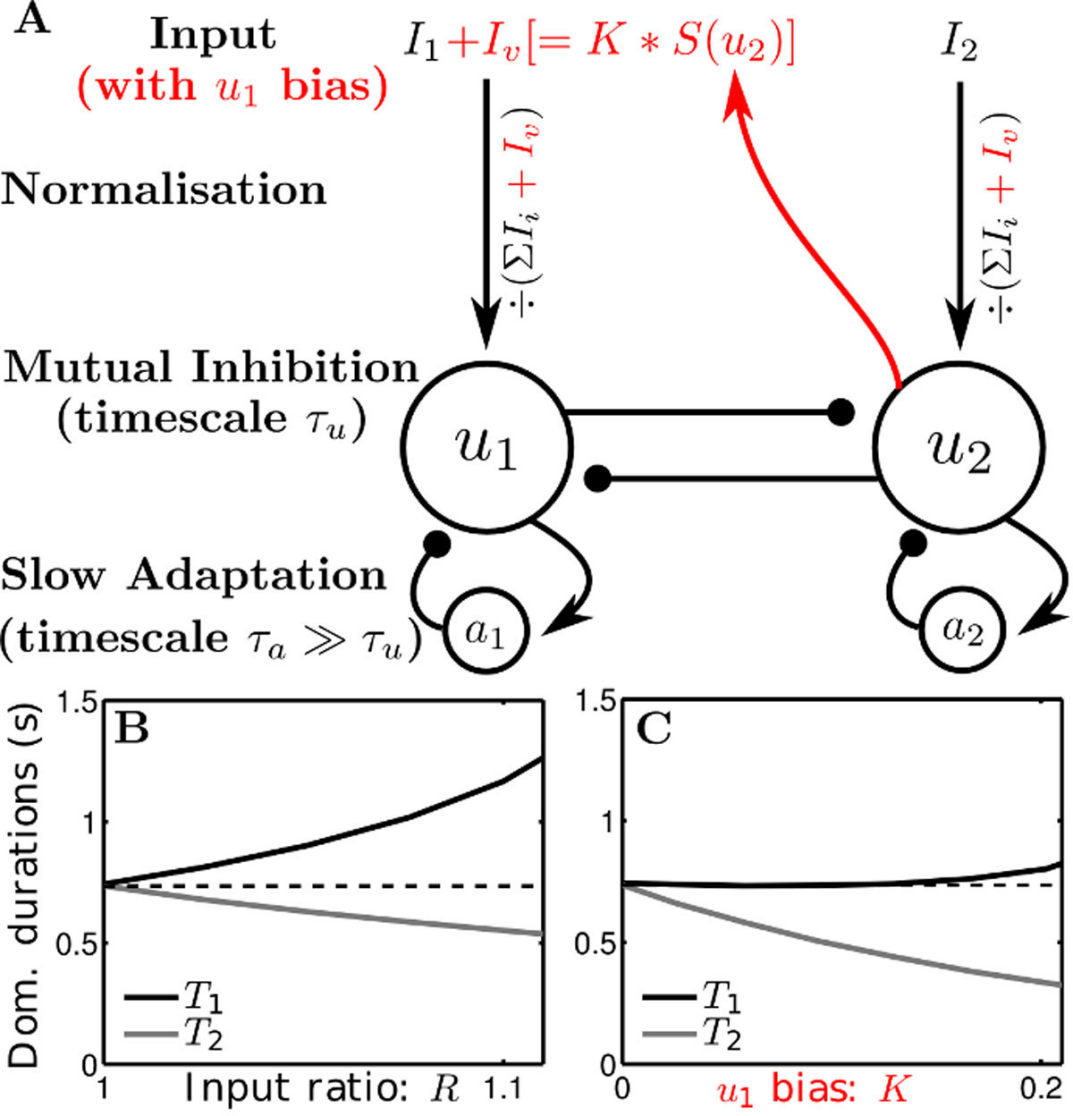
**A** Model schematic: u1 and u2 are competing populations with inputs I1, I2 and adaptation variables a1, a2. Volitional bias Iv with strength K is active when u2 active (controlled by a threshold function S). **B** Durations for u1 active (T1) and for u2 active (T2) are plotted varying the input ratio R=I1/I2 without bias (K=0). **C** As **B** but varying the u1 bias strength with input ratio R fixed at R=1.

The underlying concept of our proposed mechanism for volitional control is that when, say, percept #1 is given a bias, population #1 will receive additional input, but only when the competing #2 is active (Fig 1A, red). We assume that this volitional bias is active only when the competing population’s activity is above some threshold. We find a qualitatively different relationship (than in Levelt II) between volitional bias and the durations. Notably durations for the percept receiving volitional bias are unchanged, whereas, the durations for the weaker percept drop significantly.These results are consistent with [2] for experimental conditions where subjects exert volitional towards a particular percept.

## Conclusions

We have proposed a volitional control mechanism that resolves the apparent conflict between generalized Levelt II and the results described in [2]. Our modelling study accounts for differences between direct input strength manipulations and top-down attention that could generalize across sensory modalities.
